# Better results of finger fractures with wide awake surgery and early protected motion

**DOI:** 10.1186/1753-6561-9-S3-A48

**Published:** 2015-05-19

**Authors:** Donalod Lalonde

**Affiliations:** 1 Department of Plastic and Reconstructive Surgery, Dalhousie University, Saint John, New Brunswick, Canada, E2K 1J5

## 

The wide-awake surgical reduction of finger fractures permits intraoperative patient active movement assessment during and after K-wire closed reduction. Patients are usually cooperative when it is explained to them that surgeons can do a better job if they see the patient move the fracture during the surgery.

An open approach to fractures requires substantial soft tissue dissection. Within the open wound the skin, gliding tendons, bone and joint structures are exposed to the postoperative scarring and callus formation that sticks everything together. The blood that fills the surgical wound creates more scarring and callus which makes it even worse, especially if a tourniquet is used for the surgery, because of the tourniquet let down bleeding. All the foreign materials left in the wound, such as plates, occupy space, hindering tendon and joint movement. In addition, scar always forms over the plate and can be 1 to 2 mm thick. Each of these processes contributes to limitations of tendon and joint mobility.

For the above reasons, we prefer to reduce most finger fractures without opening them if possible. We use percutaneous K wires with mini low radiation C arms. No X-ray technicians are required to operate these low radiation X-ray machines which the surgeons operate outside the main operating room in our minor surgery clinic rooms under WALANT (Wide Awake Local Anesthesia No Tourniquet). If we do have to open the skin to dissect the fracture, at least tourniquet free epinephrine hemostasis has a much drier field after the surgery for less scarring and getting stuck.

When a finger is treated with closed reduction and K-wire fixation, the main advantage isthat there is no scarring from surgical dissection.There is reduced space for postoperative internal bleeding to accumulate and evolve into callusand scar formation. In addition, there is no permanent internal hardware to occupy space and restrict motion. K wires also provide functionally stable fixation. Although not rigid, this type of fixation allows bone to heal in a good position of function so that gliding and a good range of motion is achieved.

The major concern about K-wire fixation for finger fractures is that the fixation is not strong enough to allow early movement. Early protected movement for finger fractures is important becausea stiff finger is a useless finger. The concerns that surgeons have traditionally had with regard to early protected movement with K-wire fixated finger fractures are (1) fear of loss of reduction and (2) skin irritation/infections generated by K wires. We believe that both these concerns can be minimized, allowing the benefits of K wires and early protected motion.

After the procedure, the patient’s hand is immobilized in a splint. We counsel the patients during the surgery that the hand will be “on strike” with no finger movement and it should be elevated above the heart for the first 2 to 4 days until the swelling is gone.

This time of immobility allows the internal wounds from the K-wire insertion to clot, avoiding further internal bleeding, and allows the pain to improve enough to allow cessation of pain medications.

We begin early protected movement only when the patient has stopped all painkillers, including ibuprofen and acetaminophen (usually 2 to 4 days after K wiring). After the discontinuation of pain medications, the patient is allowed to gently move the finger while stabilizing the fracture with the fingers of the other hand under the guidance of our hand therapists who also build the appropriate splints, as they do for flexor tendon repair. The patient needs to keep the joints moving enough so the tendons and joints do not get stuck. Even a small amount of movement keeps the joints and tendons gliding. This gentle movement is predicated on the following instruction: do not do anything that hurts.

The pain of the fracture and K-wire skin irritation will stop the patient from moving the fracture or getting K wire infections. The hand is splinted at all times when not doing early protected movement.

The K wires are generally left in place for an average of 2 to 3 weeks. When the fracture is not tender to firm palpation between a thumb and index finger, the K wires can usually be removed; this is a clinically healed fracture. Radiological healing lags behind clinical healing for finger fractures and should not be used as a main guide.

